# Alteration of Tremor Dominant and Postural Instability Gait Difficulty Subtypes During the Progression of Parkinson's Disease: Analysis of the PPMI Cohort

**DOI:** 10.3389/fneur.2019.00471

**Published:** 2019-05-07

**Authors:** Jeong Won Lee, Yoo Sung Song, Hyeyun Kim, Bon D. Ku, Won Woo Lee

**Affiliations:** ^1^Department of Nuclear Medicine, International St. Mary's Hospital, Catholic Kwandong University College of Medicine, Incheon, South Korea; ^2^Department of Nuclear Medicine, Seoul National University Bundang Hospital, Seongnam, South Korea; ^3^Department of Neurology, International St. Mary's Hospital, Catholic Kwandong University College of Medicine, Incheon, South Korea; ^4^Medical Research Center, Institute of Radiation Medicine, Seoul National University, Seoul, South Korea

**Keywords:** Parkinson's disease, tremor dominant, postural instability gait difficulty, PPMI, I-123 FP-CIT SPECT

## Abstract

**Background:** Classifying PD into tremor dominant (TD) and postural instability gait difficulty (PIGD) subtypes may have several limitations, such as its diagnostic inconsistency and inability to reflect disease stage. In this study, we investigated the patterns of progression and dopaminergic denervation, by prospective evaluation at regular time intervals.

**Methods:** 325 PD dopamine replacement drug-naïve patients (age 61.2 ± 9.7, M:F = 215:110) were enrolled. Patients were grouped into TD, indeterminant, and PIGD subtypes. Clinical parameters and I-123 FP-CIT SPECT images of each groups were analyzed and compared at baseline, 1, 2, and 4 years of follow up periods.

**Results:** Baseline I-123 FP-CIT uptakes of the striatum were significantly higher in the TD group compared with the indeterminant group and PIGD group (*p* < 0.01). H & Y stage and MDS-UPDRS part III scores of the indeterminant group were significantly worse at baseline, compared with the TD and PIGD groups (*p* < 0.001 and *p* < 0.01, respectively), and MDS-UPDRS part II scores of the indeterminant group were significantly worse than the PIGD group (*p* < 0.001). There were no other significant differences of age, gender, weight, duration of PD, SCOPA-AUT, MOCA, usage of dopamine agonists, and levodopa equivalent daily doses at baseline. After 4 years of follow up, there were no differences of I-123 FP-CIT uptakes or clinical parameters, except for the MDS-UPDRS part II between the TD and indeterminant group (*p* < 0.05). The motor-subtypes were reevaluated at the 4 years period, and the proportion of patients grouped to the PIGD subtype increased. In the reevaluated PIGD group, MDS-UPDRS part II score (*p* < 0.001), SCOPA-AUT (*p* < 0.001), the proportion of patients who developed levodopa induced dyskinesia were higher than the reevaluated TD group, and the striatal I-123 FP-CIT uptakes were significantly lower (*p* < 0.01).

**Conclusion:** There are no significant differences of symptoms and dopaminergic innervation between the TD and PIGD group after a certain period of follow up. Significant portion of patients switched from the TD subtype to the PIGD subtype during disease progression, and had a worse clinical prognosis.

## Introduction

Parkinson's disease (PD) is a neurodegenerative disorder that is mainly known for the deterioration of one's motor functions. However, it is also accompanied by a broad spectrum of non-motor symptoms such as cognitive decline, mood disorder, sleep disorder, and autonomic dysfunction (1), and each PD patient presents with heterogenous symptoms and progression rates (2). Due to the various and complex manifestations of PD, the pathophysiology of each respective symptoms has been one of the major fields of investigation. For instance, the progression of gait disturbance is known to be associated with nigrostriatal dopaminergic denervation, while the progression of other non-motor symptoms such as autonomic dysfunction and cognitive deterioration is known to be associated with extrastriatal pathologies (3). Thus, identifying the homogenous groups of PD and categorizing into subtypes have been of interest for decades.

Previous studies have proposed that classifying PD into homogenous motor subtypes would be of value, by providing a better understanding of the pathology, prognosis, progression pattern, and the key to develop proper treatment strategies (4). Analysis of the motor symptoms came to define the subtypes of PD into the tremor dominant (TD) type and the postural instability gait difficulty (PIGD) type (5), and the clinical outcomes of the two subtypes have been investigated. However, there are some controversies on this topic, while some report the TD subtype to have a better prognosis and mild disease progression rate compared with the PIGD subtype, while others claim no differences of long term outcomes (6–15). Regarding to pathologies, several studies have depicted different patterns of striatal dopaminergic denervation between the two subtypes (16, 17), and some studies investigated the patterns of dopamine denervation in different motor subtypes of PD, via [I-123] N-ω-fluoropropyl- 2β-carbomethoxy- 3β-(4-iodophenyl) nortropane (I-123 FP-CIT) SPECT imaging. However, they have also have failed to show a consistent description. Some described higher striatal I-123 FP-CIT uptake in the TD subtype, while some described no significant differences between the TD and PIGD subtypes (18–21). Therefore, there still remains several unclarified issues on the progression and dopaminergic denervation of PD, in a subtype-based aspect.

The aim of our study was to compare the progressive pattern and dopaminergic denervation of PD between different subtypes, by evaluating the clinical symptoms, and I-123 FP-CIT SPECT images at regular time intervals. Loss of dopaminergic innervation and the severity of symptoms are known to worsen throughout the lifetime in a non-linear, exponential pattern (22, 23). Therefore, the functional decline and disease progression should be investigated in a prospective approach. In our study, patients were periodically evaluated in a prospective PD cohort, under the assumption that different subtypes may have different progression patterns and paces. Clinical parameters and I-123 FP-CIT SPECT image findings were evaluated and quantified upon a subgroup-based analysis, on a regular basis at baseline, 1, 2, and 4 years of follow up. Finally, the clinical subtypes of the whole group of patients were reevaluated after 4 years of follow up, to observe any changes of main motor phenotypes.

## Materials and Methods

### Patients

Subject data were downloaded from the Parkinson's Progression Markers Initiative (PPMI) database (http://www.ppmi-info.org) in April, 2018. Three hundred and twenty-five PD patients (age 61.2 ± 9.7, M:F = 215:110) were enrolled. The inclusion criteria were as follows: a diagnosis of PD for 2 years or less at the time of screening, dopamine transporter (DAT) deficit on baseline I-123 FP-CIT SPECT images, age 30 years or more, Hoehn and Yahr (H & Y) stage I or II at baseline. Patients had at least two or more I-123 FP-CIT SPECT images acquired during follow up at 1, 2, and 4 years of time points. Movement Disorder Society-Unified Parkinson's disease rating scale (MDS-UPDRS) scores, H & Y stages, levodopa equivalent daily doses (LEDD), Scale for Outcomes in Parkinson's disease-Autonomic (SCOPA-AUT), and Montreal Cognitive Assessment (MOCA) were evaluated at baseline, 1, 2, and 4 years of follow up. Patients on any kinds of PD related medications on baseline were excluded. Patients were asked to withhold their PD related medication for 12 h before motor assessment. The patient group was subdivided into tremor dominant (TD), indeterminant, and postural instability/gait difficulty (PIGD) subtypes according to TD and PIGD scores based on UPDRS items. In short, the ratio of the mean tremor scores to the mean PIGD scores was used to define TD patients (ratio ≥1.5), PIGD patients (ratio ≤1), and indeterminate patients (ratios >1.0 and <1.5) (5). All methods were performed in accordance with the relevant guidelines and regulations. Written informed consent for all clinical data were obtained of all PPMI participants, and all subjects gave written informed consent in accordance with the 1964 Declaration of Helsinki and its later amendments. The study was approved in all participating sites, respectively, by each local Institutional review boards.

### I-123 FP-CIT SPECT Analysis

I-123 FP-CIT SPECT scans were acquired 4 ± 0.5 h after I-123 FP-CIT injection (111–185 MBq). Images were reconstructed iteratively, without any filtering. In order to maintain a uniform dataset from multiple institutions, the core imaging lab of PPMI performs quality controls, such as phantom studies, validation of acquisition protocols, and standardization of image processing procedures. Respective institutions also received technical setup visits from the core imaging lab before study enrollment. Image analysis were done with the PMOD software (PMOD Technologies, Zurich, Switzerland). Specific binding ratios [SBRs, (target region/reference region)-1] of each caudate and putamen were acquired with the occipital cortex as a reference tissue. Minimum SBR values among the bilateral striatal regions were selected for analysis.

### Statistical Analysis

Medcalc version 18.9.1 (MedCalc Software, Belgium) was used for analysis. Demographic factors and striatal SBRs between the groups were compared by one-way ANOVA or Kruskal-Wallis test.

## Results

### Demographic Characteristics

Among our cohort of 325 patients, 221 patients were classified as TD (68.0%), 29 patients as indeterminant type (8.9%), and 75 patients as PIGD type (23.1%) at baseline. Baseline demographic and clinical characteristics are presented in Table 1. H & Y stage and MDS-UPDRS part III scores of the indeterminant group were significantly worse at baseline, compared with the TD and PIGD groups (*P* < 0.001 and *P* < 0.01, respectively), and MDS-UPDRS part II scores of the indeterminant group were significantly worse than the PIGD group (*P* < 0.001). The SBRs of the caudate and putamen were significantly lower in the indeterminant group and PIGD group compared with the TD group (*P* < 0.01 for both caudate and putamen). No significant differences were observed in age, gender, weight, duration of PD, SCOPA-AUT, MOCA, usage of dopamine agonists, and LEDD between any groups.

**Table 1:** Baseline patient characteristics.

	**TD subtype (*n* = 221)**	**Indeterminant subtype (*n* = 29)**	**PIGD subtype (*n* = 75)**	***P*-value**
Age at PD onset (years)	61.2 ± 9.6	62.2 ± 9.1	60.5 ± 10.1	0.71
Gender (Male: Female)	144: 78	23: 6	48: 17	0.15
Weight (kg)	82.2 ± 17.8	82.8 ± 12.7	79.5 ± 16.6	0.51
Duration of symptoms until study enroll (months)	24.7 ± 23.4	29.4 ± 21.6	18.7 ± 12.4	0.05
Caudate SBRs	1.9 ± 0.5^a^	1.6 ± 0.3^b^	1.7 ± 0.5^b^	<0.01
Putaminal SBRs	0.7 ± 0.2^a^	0.6 ± 0.1^b^	0.6 ± 0.2^b^	<0.01
H&Y staging	1.5 ± 0.5^a^	1.9 ± 0.4^b^	1.6 ± 0.5^a^	<0.001
MDS-UPDRS Part II score	5.9 ± 3.6^a^	9.4 ± 4.5^b^	8.0 ± 4.3^b^	<0.001
MDS-UPDRS Part III score	20.1 ± 8.6^a^	25.2 ± 8.9^b^	20.1 ± 8.0^a^	<0.01
SCOPA-AUT	8.4 ± 5.8	10.6 ± 5.0	9.4 ± 5.9	0.09
MOCA	27.2 ± 2.2	26.3 ± 2.7	27.2 ± 2.4	0.14
Use of dopamine agonist, %	56.5	51.7	61	0.63
Average LEDD at 48 months (g)	1264.0 ± 1288.8	1377.7 ± 1414.8	1264.2 ± 1191.6	0.90

*For a particular variable, values with different superscripts indicate statistically significant difference*.

### Serial Changes of SBRs During 4 Years of Follow Up

Caudate SBRs of the indeterminate and PIGD groups were significantly lower than that of the TD group until 1 year follow up (*P* < 0.01), and at 2 years of follow up the difference was significant in between the PIGD group and TD group only (*P* < 0.01). There were no significant differences between any groups at 4 years of follow up. Putaminal SBRs of the PIGD group were significantly lower than that of the TD group until 1 year follow-up (*P* < 0.05), and there was a significant difference between the PIGD group and TD group, at 2 years of follow up (*P* < 0.05). There were no significant differences between any groups at 4 years of follow up (Table 2).

**Table 2:** SBRs of I-123 FP-CIT during follow up.

	**TD subtype**	**Indeterminant subtype**	**PIGD subtype**	***P*-value**
**CAUDATE**				
Baseline	1.9 ± 0.5^a^ (221)	1.6 ± 0.3^b^ (29)	1.7 ± 0.5^b^ (75)	<0.01
1 year	1.7 ± 0.5^a^ (202)	1.4 ± 0.4^b^ (25)	1.5 ± 0.5^b^ (67)	<0.01
2 years	1.6 ± 0.5^a^ (196)	1.3 ± 0.4 (28)	1.4 ± 0.5^b^ (70)	<0.01
4 years	1.4 ± 0.5 (173)	1.1 ± 0.4 (22)	1.2 ± 0.5 (64)	<0.05
**PUTAMEN**				
Baseline	0.7 ± 0.2^a^ (221)	0.6 ± 0.1^b^ (29)	0.6 ± 0.2^b^ (75)	<0.01
1 year	0.6 ± 0.2^a^ (202)	0.5 ± 0.2 (25)	0.5 ± 0.2^b^ (67)	<0.05
2 years	0.6 ± 0.2^a^ (196)	0.5 ± 0.2^b^ (28)	0.5 ± 0.2 (70)	<0.05
4 years	0.5 ± 0.2 (173)	0.4 ± 0.2 (22)	0.4 ± 0.2 (64)	<0.05

*For a particular variable, values with different superscripts indicate statistically significant difference. The values in parentheses represent the number of patients*.

### Serial Changes of Clinical Parameters During 4-Years of Follow Up

Serial changes of the clinical parameters of each group were analyzed at baseline, 1, 2, and 4 years of follow up (Table 3). H & Y stage of the indeterminant group was significantly higher than the TD and PIGD groups at 2 years of follow up (*P* < 0.01), but there were no significant differences at 4 years of follow up. MDS-UPDRS part II scores of the indeterminant group were significantly higher than the TD group until 4 years of follow up (*P* < 0.05), but there were no significant differences of the MDS-UPDRS part III scores between any groups at 4 years of follow up. The SCOPA-AUT of the indeterminant group was significantly higher than the TD group at 1 year follow up, but there were no significant differences of SCOPA-AUT between any groups at 2 and 4 years of follow up. There were no significant differences of MOCA scores between any groups at throughout the follow up. There was a significantly higher portion of LID development in the indeterminant group after 4 years of follow up, with a higher score of MDS-UPDRS part IV question 4.1 (time spent with dyskinesias) (Table 4).

**Table 3:** Clinical parameters during follow up.

	**TD subtype**	**Indeterminant subtype**	**PIGD subtype**	***P*-value**
**H &Y STAGE**				
Baseline (325)	1.5 ± 0.5^a^	1.9 ± 0.4^b^	1.6 ± 1.5^a^	0.001
1 year (325)	1.7 ± 0.5	1.9 ± 0.3	1.7 ± 0.6	0.09
2 years (325)	1.7 ± 0.5^a^	2.0 ± 0.5^b^	1.8 ± 0.6^a^	<0.01
4 years (325)	1.8 ± 0.5	2.0 ± 0.5	2.0 ± 0.8	0.06
**MDS- UPDRS II**				
Baseline	5.9 ± 3.6^a^ (221)	9.4 ± 4.5^b^ (29)	8.0 ± 4.3^b^ (75)	<0.001
1 year	7.5 ± 4.6^a^ (203)	11.0 ± 4.8^b^ (26)	9.0 ± 4.5 (70)	<0.001
2 years	8.1 ± 4.7^a^ (206)	11.9 ± 5.3^b^ (28)	9.4 ± 5.6 (71)	<0.001
4 years	10.3 ± 6.0^a^ (204)	13.9± 6.2^b^ (26)	11.5 ± 7.8 (73)	<0.05
**MDS- UPDRS III**				
Baseline	20.1 ± 8.6^a^ (221)	25.2 ± 8.9^b^ (29)	20.1 ± 8.0^a^ (75)	<0.001
1 year	22.8 ± 10.1 (203)	27.8 ± 9.3 (26)	23.0 ± 10.8 (70)	0.07
2 years	24.3 ± 11.2^a^ (206)	30.0 ± 11.8^b^ (28)	26.6 ± 12.0 (71)	<0.05
4 years	29.8 ± 11.2 (182)	32.5 ± 12.4 (22)	30.9 ± 14.2 (69)	0.57
**SCOPA-AUT**				
Baseline	8.4 ± 5.8 (221)	10.6 ± 5.0 (29)	9.4 ± 5.9 (75)	0.09
1 year	9.3 ± 7.0^a^ (221)	12.6 ± 7.6^b^ (29)	11.0 ± 6.7 (75)	<0.05
2 years	12.1 ± 7.0 (206)	14.1 ± 6.1 (28)	13.1 ± 7.9 (71)	0.30
4 years	12.3 ± 7.3 (203)	16.0 ± 7.4 (26)	13.4 ± 8.5 (72)	0.05
**MOCA**				
Baseline	27.2 ± 2.2 (221)	26.3 ± 2.7 (29)	27.2 ± 2.4 (75)	0.14
1 year	26.5 ± 2.7 (220)	26.2 ± 2.8 (29)	26.5 ± 2.6 (75)	0.84
2 years	26.7 ± 2.7 (203)	25.6 ± 3.7 (28)	26.3 ± 2.6 (71)	0.13
4 years	26.8 ± 3.1 (208)	26.0 ± 4.2 (27)	26.5 ± 3.7 (73)	0.54

*For a particular variable, values with different superscripts indicate statistically significant difference. The values in parentheses represent the number of patients*.

**Table 4:** Ratio of patients affected with levodopa induced dyskinesia during follow up.

	**TD subtype**	**Indeterminant subtype**	**PIGD subtype**	***P*-value**
1 year	2% (115: 2)	6% (17: 1)	2% (48: 1)	0.39
2 years	5% (161: 9)	7% (26: 2)	7% (63: 5)	0.67
4 years	8% (177: 16)	29% (17: 7)	18% (59: 13)	<0.01
MDS-UPDRS 4.1 at 4 year	0.1 ± 0.5^a^	0.6 ± 1.2^b^	0.2 ± 0.5^a^	<0.001

*For a particular variable, values with different superscripts indicate statistically significant difference. The values in parentheses represent the number of patients without: and with levodopa induced dyskinesia*.

### Reevaluation of Clinical Subtypes After 4 Years of Follow Up

TD and PIGD scores were reevaluated after 4 years of follow up, and the patients were reevaluated into TD, indeterminant, and PIGD subtypes. The total proportion of TD group decreased from 68.0% at baseline to 44.6% at 4 years, and the proportion of PIGD group increased from 23.1% at baseline to 44.2% at 4 years. 77.8% of the indeterminant group altered to PIGD group (Table 5). Based on the reevaluated clinical subtypes, the clinical parameters at 4 years follow up were compared between each group (Table 6). MDS-UPDRS part II score, SCOPA-AUT were significantly higher in the PIGD group compared with the TD group (*P* < 0.001, both). Proportion of patients who developed levodopa induced dyskinesia was higher in the PIGD group than the TD group at 4 years of follow up, with a higher MDS-UPDRS 4.1 score (*P* < 0.01). SBRs of the caudate and putamen were significantly lower in the PIGD group than the TD group (*P* < 0.01, both).

**Table 5:** Changes of subtypes, reevaluated after 4 years of follow up.

	**TD at 4 years**	**Indeterminant at 4 years**	**PIGD at 4 years**	**Total**
TD at baseline	82 (58.3%)	18 (12.8%)	41 (29.1%)	141
Indeterminant at baseline	4 (22.2%)	0 (0%)	14 (77.8%)	18
PIGD at baseline	10 (17.8%)	6 (10.7%)	40 (71.4%)	56
Total	96	24	95	

**Table 6:** Comparison of clinical parameters based on the reevaluated subtypes after 4 years of follow up.

	**TD subtype (*n* = 96)**	**Indeterminant subtype (*n* = 24)**	**PIGD subtype (*n* = 95)**	***P*-value**
MDS-UPDRS Part II score	8.7 ± 5.2^a^	9.9 ± 5.1	12.5 ± 6.9^b^	<0.001
MDS-UPDRS Part III score	29.2 ± 10.5	31.0 ± 9.4	31.4 ± 13.7	0.49
SCOPA-AUT	11.2 ± 5.9^a^	12.7 ± 8.0	14.5 ± 8.5^b^	<0.001
MOCA	26.6 ± 3.1	26.7 ± 2.9	26.9 ± 3.4	0.76
No. of patients with LID (% of total)	6 (6.3%)	4 (16.7%)	19 (20.0%)	<0.05
MDS-UPDRS 4.1	0.1 ± 0.2^a^	0.2 ± 0.5	0.3 ± 0.8^b^	<0.01
Caudate SBRs	1.5 ± 0.5^a^	1.2 ± 0.4^b^	1.2 ± 0.3^b^	<0.01
Putaminal SBRs	0.5 ± 0.2^a^	0.4 ± 0.1	0.4 ± 0.2^b^	<0.01

*For a particular variable, values with different superscripts indicate statistically significant difference*.

## Discussion

There have been several previous studies that examined the prognoses and clinical courses of TD and PIGD subtypes. However, there are controversies on whether the clinical progression and severity of the PIGD group is worse or not, and also on whether there are any differences of the pace of dopaminergic denervation between the TD subtype and the PIGD subtype. This may be due to the relatively small number of patients, inconsistent periods of disease evaluation, and irregular timepoints of I-123 FP-CIT SPECT image acquisition. In our study, we demonstrated the sequential changes of clinical parameters and I-123 FP-CIT uptakes at baseline, and at regular periods of 1, 2, and 4 years of follow up. Additionally, we demonstrated that the clinical subtypes may also change in a large proportion, during the progression of PD. MDS-UPDRS part II scores and striatal SBRs were worse in the PIGD group than the TD group at baseline, but there were no significant differences of the H & Y staging, MDS-UPDRS part III scores, MOCA scores, MDS-UPDRS part IV question 4.1 (time spent with dyskinesias), and SCOPA-AUT at baseline between the TD group and the PIGD group. Moreover, there were no differences of any parameters between the two subtypes after 4 years of follow up. This finding corresponds to the fact that the tremor dominant feature is not related with a favorable long term prognosis (7, 13). In conclusion, our results showed no significant differences of any important clinical parameters between TD and PIGD groups after a certain period of time. This may be due to the regular periodic assessment during the follow of 4 years, but also because of the changes of motor subtypes during the progression of PD, which will be discussed in the next paragraph.

In our patient population, the proportion of PIGD patients increased after 4 years of follow up. It has been previously suggested that the subtype may change during the course of PD, and the transition of the TD subtype to the PIGD subtype suggests that motor subtypes are not different entities of PD, but are rather different stages of PD (24, 25). Our study adds evidence and several additional points of view to this stream. First of all, in regards to the progression rate, the loss of dopaminergic neurons in PD is known to progress in an exponential pattern (22, 23), which implicates a more rapid progression in the early stage of PD than the later stage. In our study, the TD subtype had higher striatal SBRs than the PIGD subtype at baseline, but the differences disappeared during the 4 years of follow up. Therefore, we also suggest that not only TD and PIGD subtypes are sequential stages of PD, but that the TD subtype has a more rapid rate of dopaminergic denervation than the PIGD subtype. Secondly, patients grouped to the TD subtype at baseline did not have a favorable prognosis. Instead, we could say that the patients who changed from the TD subtype to the PIGD subtype has a worse and faster prognosis, considering that there were no differences of symptom duration between the TD and PIGD groups prior to enrollment. Among numerous risk factors for PD, conversion of subtype may have its strength as an indicator of disease progression. Recently, it has been suggested that the conventional motor-phenotype based subtyping has several shortcomings for clinical application. As mentioned above, it is still questionable whether the subtyping is valid in predicting prognosis. Also, there may be potential confounding factors such as age, disease stage, and genetic factors that affect the reliability of subtype diagnosis at baseline (26). To overcome the limitations of TD/PIGD subtyping, recent studies performed cluster analysis with other biomarkers, and non-motor phenotypes (27, 28). According to these cluster analyses, patients grouped to the diffuse malignant subtype had a faster prognosis, but only one-third of this subtype was PIGD dominant at baseline (28). Similarly, in our study, among patients who were grouped to the PIGD subtype at 4 years, only 42% of patients were subtyped to the PIGD group at baseline. In conclusion, our findings support the suggestion that TD and PIGD are not different subtypes of PD, but are rather different clinical stages with different disease progression features. The indeterminant group may be the transitional stage from the TD group to the PIGD group, considering that the baseline striatal SBRs, H&Y stage, and MDS-UPDRS scores of the indeterminant group were worse than the TD group. Though most of the baseline indeterminant group patients (77.8%) converted to the PIGD group after 4 years, baseline H & Y staging and baseline MDS-UPDRS part III scores were worse in the indeterminant group compared with the PIGD group. This may be because of the limitation of H & Y staging, that stage II does not necessarily have a severe motor disability than stage I (29). And also, the MDS-UPDRS part III is composed of more tremor related items than posture/gait related items (5).

Unlike bradykinesia and rigidity, the severity of tremor does not correlate with striatal dopaminergic denervation (30), and has been suggested to be due to abnormal neural firing of the basal ganglia (31). Assuming from our results, tremor related pathologic changes of the basal ganglia seems to precede in the early stage of PD, followed by dopaminergic denervation of the striatum in the later stage, resulting in the conversion of dominant motor features. We have utilized the SBR values from I-123 FP-CIT SPECT images as a clinical indicator of PD progression. The degree of PD pathology is usually referred to the Braak staging, which describes the spread of Lewy bodies starting from the brain stem to the neocortex. However, the I-123 FP-CIT SPECT images do not wholly reflect the pathophysiology of PD, but show dopaminergic denervation of the striatum. We have adopted the striatal SBRs as an indirect biomarker for clinical progression, since I-123 FP-CIT SPECT image findings are known to correlate with the disease severity and duration while Braak staging does not (32–34). Though it is hard to know one's Braak stage during follow up, future studies may focus on the pathophysiology contributing to the conversion of one's phenotypes during disease progression.

There are some limitations in our study. First, the I-123 FP-CIT SPECT images could have variations since the PPMI data were collected from multiple institutions. To maintain a reliable dataset, quality control was done by the core imaging lab of PPMI, as described in the operations manual (www.ppmi-info.org). Second, our study does not include the dataset of healthy controls. The PPMI only provides the baseline clinical information of the healthy control group. By comparing the deterioration of dopaminergic innervation in normal controls, we would be able to investigate the contribution of normal aging process in the progression of PD. Finally, though we have concluded that the patient groups tended to shift to the PIGD subtype, there were still some portion of patients that were inconsistent with the tendency. 22.2 and 17.8% of the baseline indeterminant group and baseline PIGD group were reevaluated as the TD group after 4 years of follow up, respectively. The pathophysiology of PD is complex and there are many factors that can affect the subtyping. Future studies may focus on the risk factors and pathologic factors that contribute to the conversion of the subtypes, with a more sophisticated analysis of the indeterminant group.

Our study revealed that there were no significant differences of motor, autonomic dysfunction, and cognition related parameters between the TD and PIGD group after 4 years of follow up. However, this was due to the conversion of motor subtypes, resulting in a transition to a higher proportion of the PIGD subtype after 4 years. Most clinical parameters were significantly worsened in the reevaluated PIGD subtype. Therefore, instead of approaching PD as predefined TD and PIGD subtypes before treatment, it would be more reasonable to consider the conversion to the PIGD subtype as a clinical indicator for poor prognosis. Additionally, our study may give a guidance to modifying treatment strategies during the progression of PD. Some drugs such as MAO-B inhibitors have been suggested to have neuroprotective effects, but has several remaining issues to be solved, such as when it becomes effective (35, 36). Our study may contribute to future studies in applying therapeutic adjustments taking consideration into initial subtypes and subtype conversion, in order to delay disease progression.

## Data Availability

All datasets generated for this study are included in the manuscript and/or the supplementary files.

## Ethics Statement

The PPMI study was approved by the local Institutional Review Boards of all participating sites, and written informed consent for clinical and SPECT data were obtained from each participant at enrollment. All subjects gave written informed consent in accordance with the Declaration of Helsinki and its later amendments. All methods were performed in accordance with the relevant guidelines and regulations.

## Author Contributions

JL participated in analysis of data, drafting the text, and preparing the tables. YS participated in conception and design of the study, analysis of data, drafting the text, and preparing the tables. HK participated in study concept, data analysis. BK participated in study concept, data analysis. WL participated in study concept, drafting the text.

### Conflict of Interest Statement

The authors declare that the research was conducted in the absence of any commercial or financial relationships that could be construed as a potential conflict of interest.
